# Supplementary material for: Pollution and Risk Assessments of Heavy Metal(loid)s in the Soil around Lead-Zinc Smelteries via Data Integration Analysis
Source: Int J Environ Res Public Health. 2022 Aug 6;19(15):9698. doi: 10.3390/ijerph19159698 (PMC9368718; doi:10.3390/ijerph19159698)
Supplement: Supplementary file 1 [file ijerph-19-09698-s001.zip › ijerph-1821174-supplementary.pdf]

Supplementary materials

Title: Pollution and risk assessments of priority heavy metal(loid)s in the soil around lead-zinc smelteries via data integration analysis

Author: Ziruo Zhou , Chi Peng *, Xu Liu, Zhichao Jiang, Zhaohui Guo, Xiyuan Xiao

Address: School of Metallurgy and Environment, Central South University, Changsha 410083, China

*Corresponding author

E-mail address: *chipeng@csu.edu.cn*

Figures = 2

Tables = 6

List

1. **Figure S1** Heavy metal(loid) concentrations/NIPI in soils with varied sampling radius from Pb-Zn smelteries among the studies.
2. **Figure S2** Heat maps of PI^b mean values in soils near Pb-Zn smelteries among various countries.
3. **Table S1** Literature information on heavy metal(loid) concentrations in soils near Pb-Zn smelteries globally.
4. **Table S2** Classification of the geo-accumulation index for soil pollution.
5. **Table S3** Soil environment standard values in different countries.
6. **Table S4** Classification of pollution index (PI) and Nemerow integrated pollution index (NIPI).
7. **Table S5** Classification of potential ecological pollution index (ER/RI).
8. **Table S6** Correlation analysis of heavy metal(loid) concentrations in soils around Pb-Zn smelteries

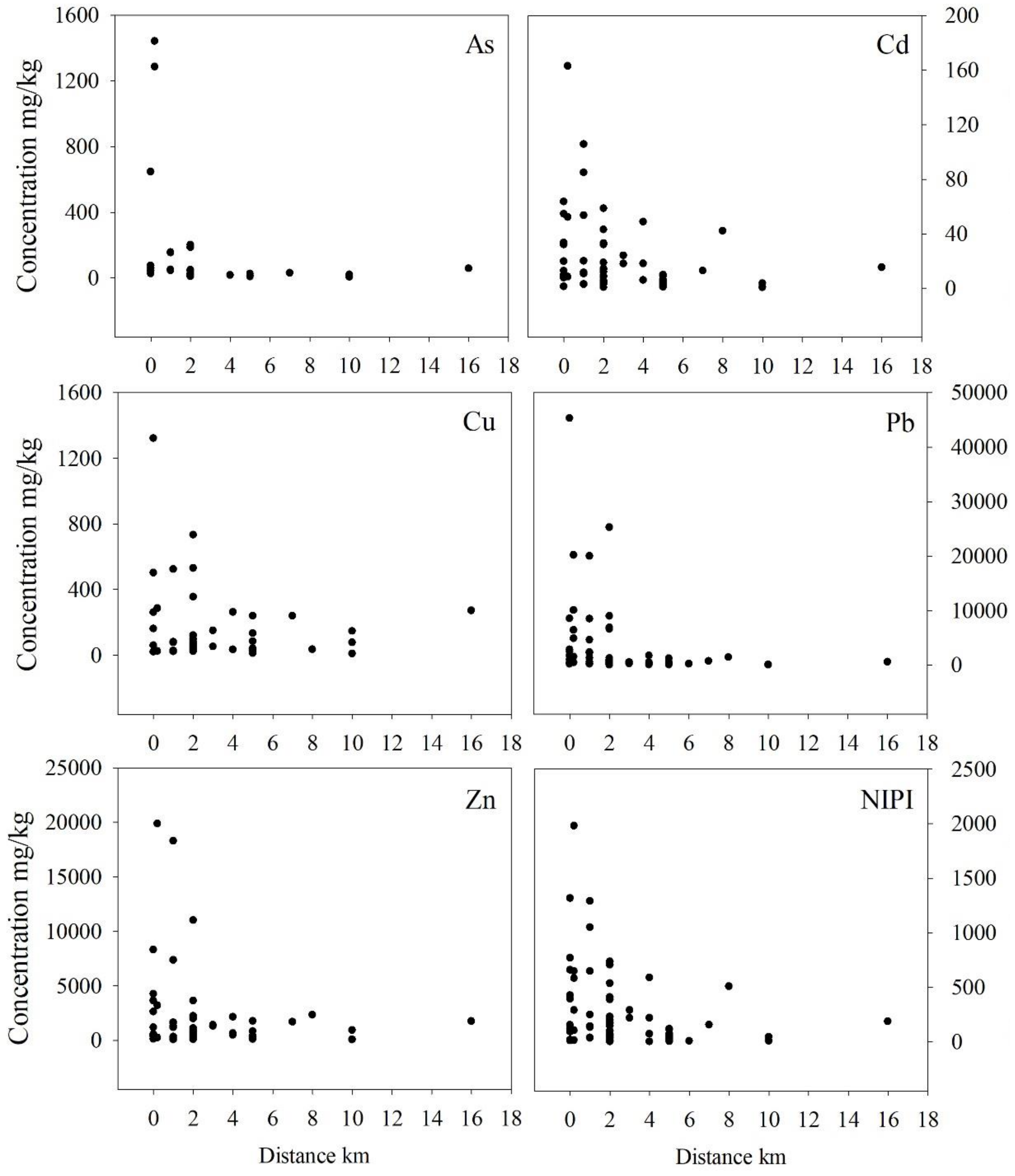

Figure S1 Heavy metal(loid) concentrations/NIPI in soils with varied sampling radius from Pb-Zn smelteries among the studies.

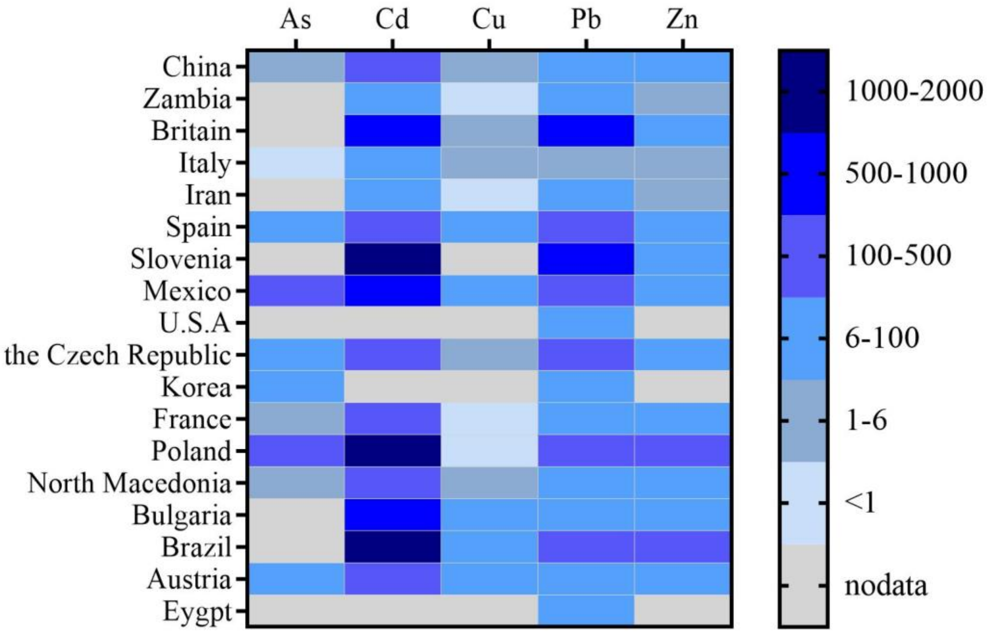

Figure S2 Heat maps of PI[b] mean values in soils near Pb-Zn smelteries among various countries.

Table S1 Literature information on heavy metal(loid) concentrations in soils near Pb-Zn smelteries globally.

Countary	City	Landuse[a]	n[b]	depth[c]	range[d]	Concentrations（mg/kg）					Reference
						As	Cd	Cu	Pb	Zn	
China	Chongqin	3	8	20	1	26.37	33.47		8528		**Zhang et al., 2018**
China	Guizhou	3	14	10	1		9.6		234	400	**Lin et al., 2009**
China	Hunan	3	9	20	1		31.89	1321	946.8	2625	**Deng et al., 2015**
China	Shanxi	3	13	10	1	43.49	63.48	260.6	2509	4244	**Liu et al., 2014**
China	Shanxi	3	9	20	1		12.72	59.14	412	556	**Liu et al., 2019**
China	Yunnan	3	4	20	1	646			2830	8304	**Ma et al., 2019**
China	Guizhou	2	22	10	1				20185		**Liu et al., 2007**
China	Guizhou	2	12	10	1				1005		**Liu et al., 2007**
China	Guizhou	2	26	10	1				453		**Liu et al., 2007**
China	Gansu	2	7	10	1		2.82	27.23	205	84.7	**Hu et al., 2015**
China	Gansu	1	4	10	1		3.06	26.88	261	67.9	**Hu et al., 2015**
China	Guangdong	2	17	20	1	44.83	2.77		211		**Luo, 2016**
China	Guangxi	2	22	5	1		11.4	21.49	532	320	**Cui et al., 2004**
China	Henan	2	20	20	1		4.96		332		**Xing et al., 2019**
China	Shanxi	2	20	20	1				58.1	115	**Liu et al., 2015**
China	Shanxi	2	17	20	1	13.3	0.7	26.9	35.2	98.6	**Xu et al., 2013**
China	Henan	2	33	20	1		5.59	23.2	144	89.6	**Gao et al., 2012**
China	Shanxi	5	15	20	1		58.5	45.3	256	3621	**Xiao et al., 2018**
China	Shanxi	5	76	20	1			31.8	41.3	102.6	**Wang et al., 2012**
China	Hunan	2	50	20	1	45.4	14.3	77.3	499	1048.3	**Li et al., 2011**
China	Hunan	1	3	20	1	24	4.1	31	209	244	**Li et al., 2011**
China	Hubei	2	101	20	2				62		**Zhou et al., 2016**
China	Fujian	5	25	20	2			84.2	1219	824	**Chen et al., 2012**
China	Shanxi	2	27	10	2	15.8	0.95	30.8	53.5	118	**Liu et al., 2015**
China	Yunnan	2	75	20	2		1.6	132	250	412	**Li et al., 2013**
China	Yunnan	2	346	20	2	25	9.1	239	512	1760	**Wu et al., 2018**
China	Yunnan	2	27	20	2	20	3.61	146	87	923	**Liu et al., 2016**
China	Yunnan	2	15	20	2	57.1	15.4	271.7	565.6	1745.1	**Liu et al., 2016**
China	Henan	2	68	20	2		3.29	31.8	184	99.2	**Xing et al., 2019**
China	Guizhou	5	7	10	2		18	52	520	1300	**Bi et al., 2006**
China	Guizhou	5	11	10	2		24	150	260	1400	**Bi et al., 2006**
China	Guizhou	5	8	10	1		43	120	9000	11000	**Bi et al., 2006**
China	Yunnan	5	23	20	2	29.9	12.8	239	712	1688	**Li et al., 2015**
Zambia	Kabwe	3	14	15	1		7.69	19.7	271.7	110.95	**Tembo et al., 2006**
Zambia	Kabwe	3	27	15	2		4.218	12.71	232.7	100.8	**Tembo et al., 2006**
Britain	Avonmouth	2	5	15	1		54.5	161	1704	3630	**Nahmani et al., 2007**
Britain	Derbyshire	3	9	15	1		19.7		45272	1173	**Li and Thornton, 2001**
Italy	Villadossola	1	18	20	2	5.15	0.63	76.87	45	75.5	**Gallini et al., 2018**
Iran	Zanjan	1	272	10	2		1.7	11.7	147.3	157.1	**Jamal et al., 2018**
Iran	Zanjan	1	21	20	2		9.82	24.8	302	311	**Ghayoraneh and Qishlaqi, 2017**

Spain	Iberian Peninsula	1	34	20	1	48.4	8.47	733	6597	424.14	**Cortada et al., 2018**
Spain	Iberian Peninsula	3	8	20	1	201	32.9	530	6877	718	**Cortada et al., 2018**
Slovenia	Žerjav	1	8	20	1		84.8		19993	1632	**Vidic et al., 2006**
Mexico	Monterrey	3	12	20	1	1442	52.1	285.4	6413	3199.7	**Gutiérrez-Ruiz et al., 2012**
U.S.A	Glover	1	4	25	1				388.4		**Prapaipong et al., 2008**
U.S.A	Glover	1	8	25	2				221.3		**Prapaipong et al., 2008**
Czekh	Pribram	2	61	5	1	153	10.6	77	2225	1180	**Rieuwerts et al., 1997**
Czekh	Pribram	2	19	5	1	156	11.4	80	2361	1237	**Rieuwerts et al., 2000**
Czekh	Trzyniec	2	1229	20	2	8.84	0.8	8.47	39.8	62.47	**Loska et al., 2004**
Czekh	Pribram	1	8	23	1	186	9.185	354.5	25270	358.5	**Komarek et al., 2007**
Korea	Chungcheongnam-do	3	153	20	1	74.5			196.9		**Kim et al., 2019**
France	Limoges	3	3	20	1		8.4	24.4	1515	244	**Michalkova et al., 2014**
France	Auby	4	26	20	1		53.4		1300	7357	**Dumoulin et al., 2017**
France	Mortagne	2	10	25	1		5.71		362.8		**Douay et al., 2007b**
France	Mortagne	4	10	25	1		2.64		323.23		**Douay et al., 2007b**
France	Calais	2	15	25	1	25.79	18.85	96.24	1274	2220	**Douay et al., 2007a**
France	Calais	4	12	25	1	21.16	13.88	73.96	880	1987	**Douay et al., 2007a**
France	Viviez	2	4	20	2					392	**Sivry et al., 2008**
France	Lille	2	31	20	2	16.2	18.07	33.8	522.7	644.6	**Sterckeman et al., 2002**
France	Lille	2	15	20	1	8.7	8.25	23.4	167.2	971	**Sterckeman et al., 2002**
France	Calais	2	36	25	2		5.9		279	486	**Pelfrene et al., 2011**
Poland	Piekary Slaskie	2	20	15	1	1286	163		4876	19859	**Siebielec et al., 2018**
Poland	Olkusz	1	12	10	2		42.03	34.6	1414	2333	**Tosza et al., 2010**
North Macedonia	Veles	4	54	5	1	13	12	52	340	460	**Stafilov et al., 2010**
North Macedonia	Veles	3	43	5	1	15	32	69	800	1100	**Stafilov et al., 2010**
North Macedonia	Veles	2	79	5	2	7.8	6.1	38	170	210	**Stafilov et al., 2010**
North Macedonia	Veles	1	26	5	2	11	4.8	41	140	180	**Stafilov et al., 2010**
Bulgaria	Kuklen	5	50	20	2		48.67	262	1738	2138	**Bacon and Dinev, 2005**
Brazil	Bahia	3	8	20	1		105	523	8472	18276	**Niemeyer et al., 2010**
Austria	Styria	2	4	30	1	59	1.24	502	347.5		**Jelecevic et al., 2019**
Austria	Arnoldstein	2	36	15	1	49.8	20	76.6	4609	1292	**Friesl et al., 2006**
Eygpt	Cairo	4	9	2	1				458		**Menrath et al., 2015**

[a] Land use are divided into five categories, 1 refers to forest land, 2 refers to farmland ,3 refers to smelting production area,4 refers to living area,5 refers to soil sround the smelting site

[b] Number of sampling points

[c] Depth(cm) of sampling points

[d] The trace metal concentrations in soils surrounding global lead and zinc smelter are divided into two ranges: 1 refers to 0-2 km ,2 refers to 0-20 km.

Table S2 Classification of the geo-accumulation index for soil pollution.

Class	Value	Soil quality
0	$I_{geo} \leq 0$	Practically uncontaminated
1	$0 \leq I_{geo} < 1$	Uncontaminated to moderately contaminated
2	$1 \leq I_{geo} < 2$	Moderately contaminated
3	$2 \leq I_{geo} < 3$	Moderately to heavily contaminated
4	$3 \leq I_{geo} < 4$	Heavily contaminated
5	$4 \leq I_{geo} < 5$	Heavily to extremely contaminated
6	$I_{geo} \geq 5$	Extremely contaminated

Table S3 Soil environment standard values in different countries.

Country	Standard value of each land type	Standard value	The purpose of risk prevention and control	Source
China	Farmland	Screening Levels	Quality and safety of agricultural products	**MEEPRC, 2018a**
China	Living Area/Industrial Area	Screening Levels	Human health	**MEEPRC, 2018b**
Canada	Farmland/Living Area/ Industrial Area	Soil Quality Guidelines	Human health / Environmental health	**CCME, 2001**
Belgium	Farmland / Living Area / Industrial Area /Forest	Trigger Levels	Groundwater security	**Halen et al., 2004**
Japan	Farmland	Pollution Indicators	Quality and safety of agricultural products	**Zhang et al., 2020**
Czech	Farmland	Pollution Indicators	Quality and safety of agricultural products	**Zhang et al., 2020**
New Zealand	Living Area / Industrial Area	Pollution Indicators	Human health	**NZME, 2011**
U.S.A	Forest	Screening Levels	Ecological risks of terrestrial plants	**USEPA, 2005**
U.S.A	Living Area	Screening Levels	Groundwater security	**USEPA, 2001**
U.S.A	Industrial Area	Screening Levels	Groundwater security	

Table S4 Classification of pollution index (PI) and Nemerow integrated pollution index (NIPI).

Class	Value	Soil quality	Value	Soil quality
0	PI ≤ 1	low degree	NIPI ≤ 0.7	Safe
1	1 ≤ PI < 3	moderate degree	0.7 ≤ NIPI < 1	Precaution
2	3 ≤ PI < 6	considerable degree	1 ≤ NIPI < 2	Slight Pollution
3	PI ≥ 6	very high degree	2 ≤ NIPI < 3	Moderate Pollution
4			NIPI ≥ 3	Heavy Pollution

Table S5 Classification of potential ecological pollution index (ER/RI).

Class	Value	Value	Soil quality
0	ER ≤ 40	RI ≤ 98	none
1	40 ≤ ER < 80	98 ≤ RI < 196	general
2	80 ≤ ER < 160	196 ≤ RI < 392	moderate
3	160 ≤ ER < 320	RI ≥ 392	high
4	ER ≥ 320		very high

Table S6 Correlation analysis of trace metal concentrations in soils around Pb-Zn smelteries.

	As	Cd	Cu	Pb	Zn
As	1	0.835**	0.926**	0.795**	0.922**
Cd		1	0.903**	0.981**	0.973**
Cu			1	0.890**	0.941**
Pb				1	0.954**
Zn					1

References

Bacon, J.R., Dinev, N.S., 2005. Isotopic characterisation of lead in contaminated soils from the vicinity of a non-ferrous metal smelter near plovdiv, bulgaria. Environ Pollut 134, 247-255. DOI: 10.1016/j.envpol.2004.07.030.

Bi, X., Feng, X., Yang, Y., Qiu, G., Li, G., Li, F., Liu, T., Fu, Z., Jin, Z., 2006. Environmental contamination of heavy metals from zinc smelting areas in hezhang county, western guizhou, china. Environ Int 32, 883-890. DOI: 10.1016/j.envint.2006.05.010.

CCME (Canadian Council of Ministers of the Environment), 2001. Canadian soil quality guidelines for the protection of environmental and human health. DOI: http://ceqg-rcqe.ccme.ca/.

Chen, B., Qiu, H.Y., Guo, Y.N., Wang, L.Y., Wang, X., 2012. Heavy metal contamination and health risk assessment in the zinc mine set area of youxi,china. Jouenal of Xiamen University (Natural Science) in China 51, 245-250. DOI: CNKI:SUN:XDZK.0.2012-02-021.

Cortada, U., Hidalgo, M.C., Martínez, J., Rey, J., 2018. Impact in soils caused by metal(loid)s in lead metallurgy. The case of la cruz smelter (southern spain). J Geochem Explor 190, 302-313. DOI: 10.1016/j.gexplo.2018.04.001.

Cui, Y.J., Zhu, Y.G., Zhai, R.H., Chen, D.Y., Huang, Y.Z., Qiu, Y., Liang, J.Z., 2004. Transfer of metals from soil to vegetables in an area near a smelter in nanning, china. Environ Int 30, 785-791. DOI: 10.1016/j.envint.2004.01.003.

Deng, X.H., Chai, L.Y., Yang, Z.H., 2015. Study of the characteristics of heavy metal pollution at pb/zn smelting slag site. Ecology and Environmental Sciences in China 24, 1534-1539. DOI: 10.16258/j.cnki.1674-5906.2015.09.017.

Douay, F., Pruvot, C., Roussel, H., Ciesielski, H., Fourrier, H., Proix, N., Waterlot, C., 2007a. Contamination of urban soils in an area of northern france polluted by dust emissions of two smelters. Water Air Soil Poll 188, 247-260. DOI: 10.1007/s11270-007-9541-7.

Douay, F., Roussel, H., Fourrier, H., Heyman, C., Chateau, G., 2007b. Investigation of heavy metal concentrations on urban soils, dust and vegetables nearby a former smelter site in mortagne du nord, northern france. J Soil Sediment 7, 143-146. DOI:

10.1065/jss2007.02.205.

Dumoulin, D., Billon, G., Proix, N., Frérot, H., Pauwels, M., Saumitou-Laprade, P., 2017. Impact of a zinc processing factory on surrounding surficial soil contamination. J Geochem Explor 172, 142-150. DOI: 10.1016/j.gexplo.2016.10.005.

Friesl, W., Friedl, J., Platzer, K., Horak, O., Gerzabek, M.H., 2006. Remediation of contaminated agricultural soils near a former pb/zn smelter in austria: Batch, pot and field experiments. Environ Pollut 144, 40-50. DOI: 10.1016/j.envpol.2006.01.012.

Gallini, L., Ajmone-Marsan, F., Scalenghe, R., 2018. The contamination legacy of a decommissioned iron smelter in the italian alps. J Geochem Explor 186, 121-128. DOI: 10.1016/j.gexplo.2017.12.013.

Gao, X.L., Xing, W.Q., Ran, Y.L., Zhou, W.D., Li, L.P., 2012. Effects of accumulation of heavy metals in soils on enzyme activities. Asian Journal of Ecotoxicology in China 7, 331-336. DOI: CNKI:SUN:STDL.0.2012-03-017.

Ghayoraneh, M., Qishlaqi, A., 2017. Concentration, distribution and speciation of toxic metals in soils along a transect around a zn/pb smelter in the northwest of iran. J Geochem Explor 180, 1-14. DOI: 10.1016/j.gexplo.2017.05.007.

Gutiérrez-Ruiz, M.E., Ceniceros-Gómez, A.E., Villalobos, M., Romero, F., Santiago, P., 2012. Natural arsenic attenuation via metal arsenate precipitation in soils contaminated with metallurgical wastes: Ii. Cumulative evidence and identification of minor processes. Appl Geochem 27, 2204-2214. DOI: 10.1016/j.apgeochem.2012.02.021.

Halen, H., Maes, E., Moutie, R.M., 2004. Procédures de calcul des norms pour le sol: Valeur de référence (vr) ,valeur seuil (vs) et valeur d'intervention (9vi) . Rapport spaque: Document de.

Hu, X.F., Jiang, Y.F., Zhan, H.Y., Zhu, K., 2015. Study on transformation of heavy metal in soil from typical pb-zn smelting areas. Environmental Monitoring in China 31, 92-97. DOI: 10.19316/j.issn.1002-6002.2015.02.018.

Jamal, A., Delavar, M.A., Naderi, A., Nourieh, N., Medi, B., Mahvi, A.H., 2018. Distribution and health risk assessment of heavy metals in soil surrounding a lead and zinc smelting plant in zanjan, iran. Hum Ecol Risk Assess 25, 1018-1033. DOI:

10.1080/10807039.2018.1460191.

Jelecevic, A., Horn, D., Eigner, H., Sager, M., Liebhard, P., Moder, K., Vollprecht, D., 2019. Kinetics of lead release from soils at historic mining and smelting sites, determined by a modified electro-ultrafiltration. Plant Soil Environ 65, 298-306. DOI: 10.17221/611/2018-pse.

Kim, H.R., Kim, K.H., Yu, S., Moniruzzaman, M., Hwang, S., Lee, G.T., Yun, S.T., 2019. Better assessment of the distribution of as and pb in soils in a former smelting area, using ordinary co-kriging and sequential gaussian co-simulation of portable x-ray fluorescence (pxrf) and icp-aes data. Geoderma 341, 26-38. DOI: 10.1016/j.geoderma.2019.01.031.

Komarek, M., Chrastny, V., Stichova, J., 2007. Metal/metalloid contamination and isotopic composition of lead in edible mushrooms and forest soils originating from a smelting area. Environ Int 33, 677-684. DOI: 10.1016/j.envint.2007.02.001.

Li, J.Y., Yang, Y.Z., Li, Z.L., Chen, J.M., Jiang, Y.X., 2013. In-situ heavy-metal pollution and health risks in the vegetables in datun town, gejiu city, yunnan. Journal of Safety and Environment in China 13, 91-96. DOI: 10.3969/ j.issn.1009-6094.2013.02.020.

Li, P., Lin, C., Cheng, H., Duan, X., Lei, K., 2015. Contamination and health risks of soil heavy metals around a lead/zinc smelter in southwestern china. Ecotoxicol Environ Saf 113, 391-399. DOI: 10.1016/j.ecoenv.2014.12.025.

Li, X.D., Thornton, I., 2001. Chemical partitioning of trace and major elements in soils contaminated by mining and smelting activities. Appl Geochem 16, 1693-1706. DOI: 10.1016/s0883-2927(01)00065-8.

Li, Z.G., Feng, X.B., Li, G.H., Bi, X.Y., Sun, G.G., Zhu, J.M., Qin, H.B., Wang, J.X., 2011. Mercury and other metal and metalloid soil contamination near a pb/zn smelter in east hunan province, china. Appl Geochem 26, 160-166. DOI: 10.1016/j.apgeochem.2010.11.014.

Lin, W.J., Xiao, T.F., Zhou, W.C., Ao, Z.Q., 2009. Environmental concerns on geochemical mobility of lead□zinc and cadmium from zinc smelting areas: Western guizhou□china. Environmental Science in China 30, 2065-2070. DOI: 10.13227/j.hjkx.2009.07.030.

Liu, T.Z., Yang, Y.G., Bi, X.Y., Li, F.L., Fu, Z.Y., Jin, Z.S., 2007. The distribution characters

and chemical speciation variations of lead in the soil induced by local zinc smelting activities in hezhang county province. Earth and Environment in China 35, 123-127.

Liu, X.Y., Chen, M.B., Li, L.Z., Hu, G.C., Huang, J.H., Liu, S., Zhang, L.J., Yu, Y.J., 2016. Contaminant characteristics and health risk assessment of heavy metals in soils from lead-zincs melting plant in huize county, yunnan province, china. Journal of Agricultural Resources and Environment in China 33, 221-229. DOI: 10.13254/j.jare.2015.0306.

Liu, Y., Wang, C.J., Feng, T., 2014. Contamination assessment and spatial distribution of heavy metals in lead-zinc smelting plant. Journal of Northwest University (Natural Science Edition) in China 44. DOI: 10.16152/j.cnki.xdxbzr.2014.01.021.

Liu, Y., Wang, C.J., Liu, H.Y., Ma, H.Z., Zhang, Q.H., Feng, T., Sun, D.L., 2015. Spatial distribution and ecological risk assessment of heavy metals in soil around a lead and zinc smelter. Chinese Journal of Environmental Engineering 9, 477-484.

Liu, Z.F., Hu, S.B., Song, F.M., 2019. Pollution characteristics and speciation analysis of heavy metals in soils around a lead-zinc smelter area in shaanxi province， china. Journal of Agro-Environment Science 38, 818-826. DOI: 10.11654/jaes.2018-1097.

Loska, K., Wiechuła, D., Korus, I., 2004. Metal contamination of farming soils affected by industry. Environ Int 30, 159-165. DOI: 10.1016/s0160-4120(03)00157-0.

Luo, Y.H., 2016. Investigation of soil pollution from heavy metals and assessment of its ecological risk around a smelting plant in shaoguan. Journal of Anhui Agri. Sci . in China 44, 133-136. DOI: 10.13989/j.cnki.0517-6611.2016.19.044.

Ma, J., Lei, M., Weng, L., Li, Y., Chen, Y., Islam, M.S., Zhao, J., Chen, T., 2019. Fractions and colloidal distribution of arsenic associated with iron oxide minerals in lead-zinc mine-contaminated soils: Comparison of tailings and smelter pollution. Chemosphere 227, 614-623. DOI: 10.1016/j.chemosphere.2019.04.030.

MEEPRC (Ministry of Ecology and Environment of the People's Republic of China), 2018a. Soil environmental quality-risk control standard for soil contaminate of agricultural land,

MEEPRC (Ministry of Ecology and Environment of the People's Republic of China), 2018b. Soil environmental quality-risk control standard for soil contaminate of development land.

Menrath, W., Zakaria, Y., El-Safty, A., Clark, C.S., Roda, S.M., Elsayed, E., Lind, C., Pesce, J., Peng, H., 2015. Use of a field portable x-ray fluorescence analyzer for environmental exposure assessment of a neighborhood in cairo, egypt adjacent to the site of a former secondary lead smelter. J Occup Environ Hyg 12, 555-563. DOI: 10.1080/15459624.2015.1020382.

Michalkova, Z., Komarek, M., Sillerova, H., Della Puppa, L., Joussein, E., Bordas, F., Vanek, A., Vanek, O., Ettler, V., 2014. Evaluating the potential of three fe- and mn-(nano)oxides for the stabilization of cd, cu and pb in contaminated soils. J Environ Manage 146, 226-234. DOI: 10.1016/j.jenvman.2014.08.004.

Nahmani, J., Hodson, M.E., Black, S., 2007. Effects of metals on life cycle parameters of the earthworm eisenia fetida exposed to field-contaminated, metal-polluted soils. Environ Pollut 149, 44-58. DOI: 10.1016/j.envpol.2006.12.018.

Niemeyer, J., Moreira-Santos, M., Nogueira, M., Carvalho, G.M., Ribeiro, R., Da Silva, E.M., Sousa, J.P., 2010. Environmental risk assessment of a metal-contaminated area in the tropics. Tier i: Screening phase. J Soils Sediments 10, 1557-1571. DOI: 10.1007/s11368-010-0255-x.

NZME (New Zealand Ministry of the Environment), 2011. Proposed national environmental standard for assessing and managing contaminants in soil to protect human health,

Pelfrene, A., Waterlot, C., Mazzuca, M., Nisse, C., Bidar, G., Douay, F., 2011. Assessing cd, pb, zn human bioaccessibility in smelter-contaminated agricultural topsoils (northern france). Environ Geochem Health 33, 477-493. DOI: 10.1007/s10653-010-9365-z.

Prapaipong, P., Enssle, C.W., Morris, J.D., Shock, E.L., Lindvall, R.E., 2008. Rapid transport of anthropogenic lead through soils in southeast missouri. Appl Geochem 23, 2156-2170. DOI: 10.1016/j.apgeochem.2008.05.002.

Rieuwerts, J.S., Farago, M., CikrtI, M., Bencko, V., 1997. Heavy metal concentrations in and around households near a secondary lead smelter. Environ Monit Assess 58, 317-335. DOI: 10.1023/A:1006058331453.

Rieuwerts, J.S., Farago, M., CikrtI, M., Bencko, V., 2000. Differences in lead bioavailablity between a smelting and a mining area. Water Air Soil Poll 122, 203-229. DOI:

10.1023/A:1005251527946.

Siebielec, S., Siebielec, G., Stuczynski, T., Sugier, P., Grzeda, E., Grzadziel, J., 2018. Long term insight into biodiversity of a smelter wasteland reclaimed with biosolids and by-product lime. Sci Total Environ 636, 1048-1057. DOI: 10.1016/j.scitotenv.2018.04.372.

Sivry, Y., Riotte, J., Sonke, J.E., Audry, S., Schäfer, J., Viers, J., Blanc, G., Freydier, R., Dupré, B., 2008. Zn isotopes as tracers of anthropogenic pollution from zn-ore smelters the riou mort–lot river system. Chem Geol 255, 295-304. DOI: 10.1016/j.chemgeo.2008.06.038.

Stafilov, T., Sajn, R., Pancevski, Z., Boev, B., Frontasyeva, M.V., Strelkova, L.P., 2010. Heavy metal contamination of topsoils around a lead and zinc smelter in the republic of macedonia. J Hazard Mater 175, 896-914. DOI: 10.1016/j.jhazmat.2009.10.094.

Sterckeman, T., Douay, F., Proix, N., Fourrier, H., Perdrix, E., 2002. Assessment of the contamination of cultivated soils by eighteen trace elements around smelters in the north of france. Water Air Soil Poll 135, 173-194. DOI: 10.1023/A:1014758811194.

Tembo, B.D., Sichilongo, K., Cernak, J., 2006. Distribution of copper, lead, cadmium and zinc concentrations in soils around kabwe town in zambia. Chemosphere 63, 497-501. DOI: 10.1016/j.chemosphere.2005.08.002.

Tosza, E., Dumnicka, E., Niklińska, M., Rożen, A., 2010. Enchytraeid and earthworm communities along a pollution gradient near olkusz (southern poland). Eur J Soil Biol 46, 218-224. DOI: 10.1016/j.ejsobi.2010.04.001.

USEPA (United States Environmental Protection Agency), 2001. Supplemental guidance for developing soil screening levels for superfund sites.

USEPA (United States Environmental Protection Agency), 2005. Ecological soil screening levels for cadmium interim final.

Vidic, T., Jogan, N., Drobne, D., Vilhar, B., 2006. Natural revegetation in the vicinity of the former lead smelter in zˇerjav, slovenia. Environ Sci Technol 40, 4119-4125. DOI: 10.1021/es052339x.

Wang, L.J., Lu, X.W., Jing, Q., Ren, C.H., Chen, C.C., Li, X.X., Luo, D.C., 2012. Heavy metals pollution in soil around the lead-zinc smelting plant in changqing town of baoji

city, china. Journal of Agro-Environment Science in China 31, 325-330. DOI: CNKI:SUN:NHBH.0.2012-02-019.

Wu, J., Long, J., Liu, L., Li, J., Liao, H., Zhang, M., Zhao, C., Wu, Q., 2018. Risk assessment and source identification of toxic metals in the agricultural soil around a pb/zn mining and smelting area in southwest china. Int J Environ Res Public Health 15. DOI: 10.3390/ijerph15091838.

Xiao, R., Shen, F., Du, J., Li, R., Lahori, A.H., Zhang, Z., 2018. Screening of native plants from wasteland surrounding a zn smelter in feng county china, for phytoremediation. Ecotoxicol Environ Saf 162, 178-183. DOI: 10.1016/j.ecoenv.2018.06.095.

Xing, W., Zheng, Y., Scheckel, K.G., Luo, Y., Li, L., 2019. Spatial distribution of smelter emission heavy metals on farmland soil. Environ Monit Assess 191, 115. DOI: 10.1007/s10661-019-7254-1.

Xu, Y.X., Wang, Q.H., Xue, L., Peng, Y.K., Wang, H.B., 2013. The characteristics of heavy metals in soil around the lead and zinc smelter in western guanzhon. Chinese Journal of Soil Science 44, 1240-1244. DOI: 10.19336/j.cnki.trtb.2013.05.036.

Zhang, M., Wang, X., Liu, C., Lu, J., Qin, Y., Mo, Y., Xiao, P., Liu, Y., 2020. Identification of the heavy metal pollution sources in the rhizosphere soil of farmland irrigated by the yellow river using pmf analysis combined with multiple analysis methods-using zhongwei city, ningxia, as an example. Environ Sci Pollut Res Int 27, 16203-16214. DOI: 10.1007/s11356-020-07986-z.

Zhang, Y.L., Ding, X.M., Liu, H.L., 2018. Study on microstructure of the heavy metal contaminated soil under an abandoned lead smelting factory in chongqing. Journal of Disaster Prevention and Mitigration Engineering in China 38, 809-814. DOI: 10.13409/j.cnki.jdpme.2018.05.006.

Zhou, T.S., Pan, l.b., Wang, M.X., Liu, K., Zhong, B.Q., Zhang, J.L., 2016. Lead contents and distribution characteristics of soils and vegetables around an abandoned lead smeltery. Journal of Agricultural Resources and Environment in China 33, 376-383. DOI: 10.13254/j.jare.2016.0065.
